# Prognostic implications of myocardial fibrosis and troponin levels measured by a highly sensitive assay in non-ischemic cardiomyopathy

**DOI:** 10.1186/1532-429X-18-S1-P121

**Published:** 2016-01-27

**Authors:** Eric Y Yang, Venkateshwar Polsani, Gerd Brunner, Faisal Nabi, Ron C Hoogeveen, Vijay Nambi, Christie M Ballantyne, Dipan J Shah

**Affiliations:** 1 Medicine, Baylor College of Medicine, Houston, TX USA; 2 Piedmont Healthcare, Atlanta, GA USA; 3 Houston Methodist Hospital, Houston, TX USA; 4 Michael E. DeBakey Veterans Affairs Medical Center, Houston, TX USA

## Background

Myocardial replacement fibrosis detected as late gadolinium enhancement (LGE) on cardiac magnetic resonance imaging (CMR) and elevated serum cardiac troponin T levels measured with highly sensitive assays (hs-cTnT) have separately been shown to be associated with subsequent heart failure events in patients with non-ischemic cardiomyopathy (NICMP). Correlations between the two markers have also been reported, but limited data exist on the significance of elevated hs-cTnT levels and LGE presence in conjunction on heart failure events. We examined the association of these markers with heart failure outcomes in NICMP patients presenting to a tertiary center

## Methods

Patients who presented for CMR between April 2008 and January 2012, received intravenous gadolinium, consented for blood draw, and were diagnosed with NICMP were included. Patients were excluded for any evidence of prior myocardial infarction, severe valvulopathy, cardiac mass, or infiltrative disorder. Cardiac parameters were abstracted from available CMR reports. Blood samples were measured with a highly sensitive assay using an automated platform (Cobas-e411 Troponin T hs, Roche Diagnostics). Subsequent hospitalization for decompensated heart failure, mechanical circulatory support, or heart transplant were abstracted from the available electronic health record (EHR). Deaths were abstracted from the EHR and the Social Security Death Index.

## Results

**Figure 1:**
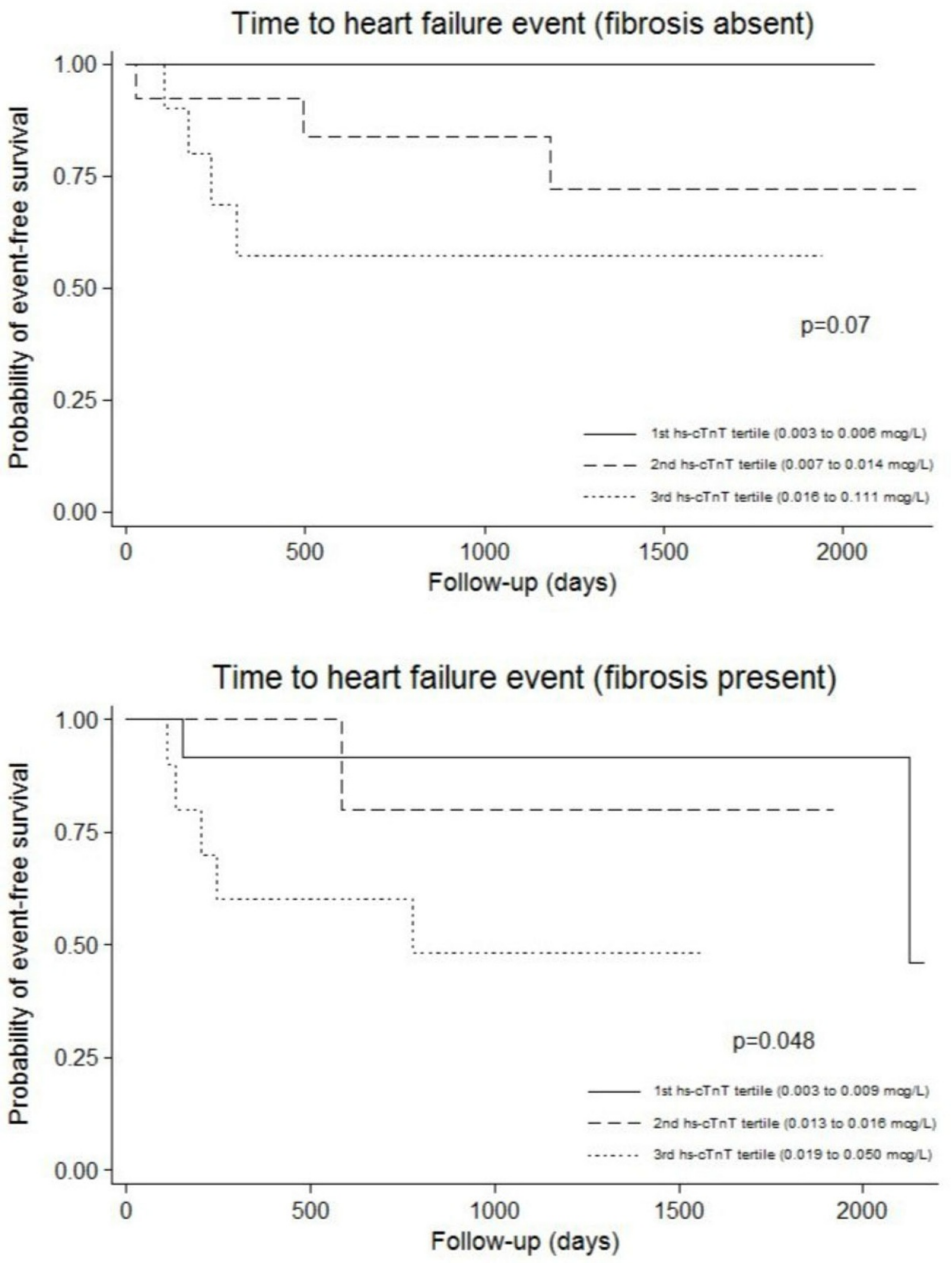
**Kaplan-Meier event-free survival estimates from heart failure events in non-ischemic cardiomyopathy patients in the absence (top panel) and presence (bottom panel) of myocardial fibrosis on delayed enhancement imaging**. Each group of patients were sub-divided by tertiles of cardiac troponin T measured with a highly sensitive assay (hs-cTnT).

Of 98 eligible patients, 74 had follow-up data available (median follow-up 2.3 [interquartile range (IQR) 0.9, 4.3] years) (Table 1). Over the interval period, there were 15 heart failure events (3 deaths, 13 heart failure hospitalizations). In patients without LGE, there was a trend toward an association between hs-cTnT and heart failure outcomes (p = 0.07) (Figure). In patients with LGE, there was a significant association between hs-cTnT and heart failure outcomes (p = 0.048) (Figure 1).

**Table 1:** Baseline characteristics, cardiac parameters derived from magnetic resonance imaging, and serum troponin levels.

Variable*		Fibrosis Absent (n = 39)	Fibrosis Present (n = 35)
Age (years)		58 (44, 68)	58 (45, 70)
Male (%)		61.5	60.0
Race (%)	White	56.4	60.0
	Black	25.6	22.9
	Hispanic	5.1	8.6
	Asian	0	5.7
	Other	12.8	2.9
Hypertension (%)		56.8	50.0
Dyslipidemia (%)		38.9	50.0
Diabetes (%)		11.1	16.1
Smoking History (%)	Former (>1 year)	22.9	20.0
	Current	5.7	16.7
Height (ins.)		69 (66, 73)	68.5 (65.5, 70)
Weight (lbs.)		200 (162, 230)	195 (146.5, 233)
Body surface area (m^2)		2.09 (1.85, 2.30)	2.05 (1.79, 2.28)
Systolic blood pressure (mm Hg)		117 (111,141)	117 (108, 133)
Diastolic blood pressure (mm Hg)		76 (65, 81)	71 (64, 82)
Heart rate (bpm)		80 (70, 86)	74 (64, 86)
Left atrial volume (mL)		109 (87, 165)	122 (87, 179)
Left ventricular ejection fraction (%)		37 (27, 45)	35 (27, 46)
Left ventricular end-diastolic volume (mL)		200 (156, 238)	214 (170, 264)
Left ventricular end-systolic volume (mL)		127 (80, 155)	133 (88, 185)
Left ventricular stroke volume (mL)		68 (53, 79)	74 (63, 91)
Left ventricular muscle mass (gm)		127.95 (128.00, 201.40)	177.00 (132.70, 223.00)
Left ventricular fibrosis percentage (%)†		0.00 (0.00, 0.00)	3.00 (2.00, 7.00)
Troponin T (ng/L)		8.0 (4.0, 21.0)	14.0 (7.0, 21.0)

*All values are proportions or median (interquartile range).

†p <0.05 (by Chi-squared or Fisher exact for categorical variables as appropriate; Wilcoxon rank sum test for continuous variables).

## Conclusions

In patients with non-ischemic cardiomyopathy, cardiac troponin T levels measured with a highly sensitive assay are associated with heart failure outcomes in the presence of late gadolinium enhancement. These findings may support a strategy of combining LGE detection and hs-cTnT levels to risk-stratify further patients with non-ischemic cardiomyopathy.

